# Strategies To Study The Neuroscience of Alcoholism: Introduction

**Published:** 2008

Alcohol use and abuse are widespread in the U.S. population. Moreover, for each drinker, alcohol consumption, particularly at excessive levels, has a vast range of effects on the body. Accordingly, research programs aimed at understanding alcohol’s effects on the individual as well as on society are similarly varied and widespread. Much of this research focuses on alcohol’s impact on the brain and individual nerve cells (i.e., neurons). A detailed survey of the strategies used to investigate the neural mechanisms associated with alcohol use and abuse would easily fill multiple volumes. Instead, this Special Section provides brief reviews of topics largely associated with two areas of research:

- What strategies can researchers use to image the acute and chronic effects of alcohol on brain function?
- How can investigators detect the genes, gene products, and gene networks associated with alcohol-related traits?

Neither area is covered completely, but the reader is provided a reasonable sampling. In any field of investigation—and especially in the neuroscience arena—there are multiple levels of analysis that can be used. Accordingly, this section is organized using a top-down approach (see box). The approaches discussed will begin with those that look at the whole brain (typically in live subjects or in clinical settings); proceed to those that examine specific brain regions, cells, molecules, or gene products; and conclude with an examination of several examples of databases allowing researchers from anywhere in the world to access and integrate data using the Internet. The emphasis of these reviews clearly is on preclinical research, although several sections also focus on clinical applications. For many topics, the broad impact of alcohol research on molecular and behavioral neuroscience is emphasized.

The Special Section begins with a discussion of several neuroimaging approaches. Thus, the first four articles look at the use of positron emission tomography (PET^1^); analysis of the brain’s electrical activity, such as event-related potentials (ERPs); and magnetic resonance imaging and spectroscopy (MRI/MRS) for studying issues of relevance to alcohol research. These techniques especially are valuable in that they allow for noninvasive evaluation of changes as they occur in the living human or animal, often in real time.

Although there are obvious advantages to using functional, real-time imaging, and the reviewed imaging technologies can be used to gather human data, many questions cannot be answered using these techniques. For example, to examine proteins or gene products, scientists usually have to utilize animal models, which can be advantageous in many ways. Therefore, five sections will describe the use of animal models that allow for precise analysis of a given brain region or even subset of cells (i.e., laser microdissection), examine the contribution of proteins and RNA to alcohol-related behaviors (i.e., proteomics and the use of small interfering RNA [siRNA]), and enable researchers to correlate specific chromosomes or genes to alcohol-related behaviors (i.e., quantitative trait locus [QTL] mapping and the use of congenic mouse strains). Importantly, recent advances in many of these technologies now also allow investigators to use these approaches to collect human data.

In general, however, laboratories around the world investigating the genetic basis for alcoholism are exploiting differences in gene expression between brains of mice bred for either high or low alcohol consumption, using platforms (i.e., microarrays) that allow for the simultaneous analysis of hundreds or thousands of genes and gene products. These approaches already have identified some genes that may play a role in determining alcohol-related behaviors. One apparent feature of these candidate genes is their unprecedented heterogeneity. The list contains genes involved in signaling, proliferation, the copying of genetic information (i.e., transcription), enzymatic activity, and stress responses, to name a few. To evaluate such a cornucopia of genetic material for functional effects in the development of alcohol-seeking behavior, a coordinated effort and the use of the most effective methodologies are necessary. In most cases to date, animals in whom specific genes have been inactivated (i.e., knockout animals) and molecular strategies to silence specific genes (i.e., so-called antisense approaches) are being utilized to confirm the involvement of specific genes. Recently, however, strategies to silence gene expression using an approach called RNA interference (RNAi) have matured enough to address this issue as well. RNAi technology is relatively reliable, applicable to essentially any gene of any species, and is now commercially available in various forms. Importantly, this technique allows investigators to reduce (i.e., downregulate) rather than completely eliminate expression of a specific gene. Because people with genetic risk factors for addiction are unlikely to carry completely inactive variants (i.e., null alleles) of any particular gene, the RNAi approach mimics the human condition more closely than traditional knockout mice. Accordingly, two articles in this Special Section describe RNAi strategies.

Looking for alcohol-related genes using any of these molecular and genetic approaches frequently involves comparing gene expression data for multiple inbred mouse strains. However, some tools used in these strategies (e.g., gene expression arrays) are designed only for analyses with one specific mouse strain called C57BL/6J mice. The genetic differences (i.e., single-nucleotide polymorphisms [SNPs]) that differentiate other strains from the C57BL/6J strain can markedly affect the results of the analyses. Therefore, one article describes an approach for addressing this problem.

Another result of the use of new, high-throughput molecular technologies, such as gene expression arrays and others, is the accumulation of enormous amounts of data. In response to this “problem,” a new field is developing in alcohol research—bioinformatics or data mining. The development of new software, databases, and Web sites allows researchers studying gene expression not only to refine the large datasets derived at their own laboratories to produce more biologically significant results but also to compare data across platforms and laboratories, resulting in a better understanding of how an individual finding fits into the current landscape of alcohol research. Therefore, the final two articles describe two databases that have been developed specifically for researchers interested in the genetic effects of alcohol.

**Figure:**
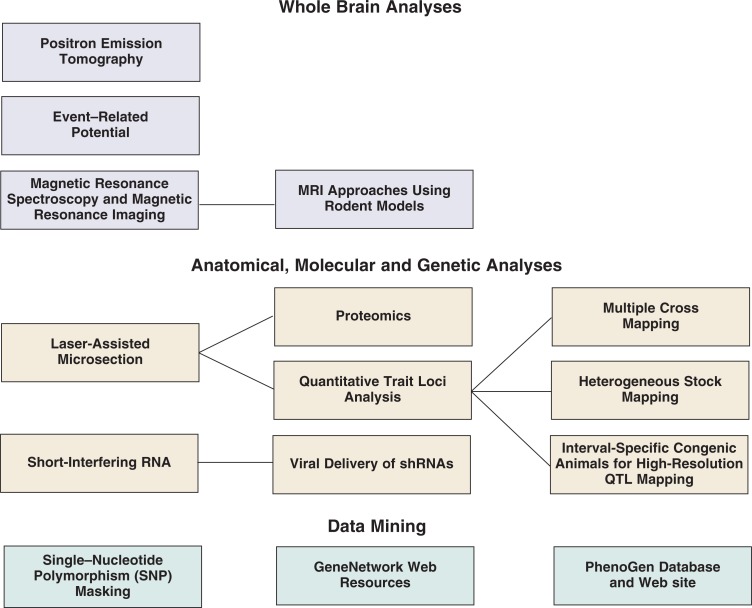
A summary of technologies covered in this Special Section. The section is arranged in a “top-down” fashion, from whole-brain analyses down to analyses of genetic products and data mining. The topics range from techniques examining whole-brain activity in awake subjects (e.g., magnetic resonance imaging [MRI]), to strategies to assess gene expression (e.g., quantitative trait loci [QTL] mapping), down to the use of bioinformatics to analyze the large amounts of data gathered with these technologies.

## Footnotes

1 For a definition of this and other technical terms, see the Glossary, pp. 279–283.

